# Effects of Four Different Dietary Fibre Supplements on Weight Loss and Lipid and Glucose Serum Profiles during Energy Restriction in Patients with Traits of Metabolic Syndrome: A Comparative, Randomized, Placebo-Controlled Study

**DOI:** 10.3390/foods12112122

**Published:** 2023-05-24

**Authors:** Zala Jenko Pražnikar, Nina Mohorko, Dejan Gmajner, Saša Kenig, Ana Petelin

**Affiliations:** 1 Faculty of Health Sciences, University of Primorska, 6310 Izola, Slovenia; zala.praznikar@fvz.upr.si (Z.J.P.); nina.mohorko@fvz.upr.si (N.M.); sasa.kenig@fvz.upr.si (S.K.); 2 Biostile Ltd., 6223 Komen, Slovenia; dejan.gmajner@biostile.si

**Keywords:** dietary fibre, obesity, weight loss, glucomannan, psyllium, inulin, β-glucans, maltodextrin

## Abstract

Obesity and its associated complications require various lifestyle changes and treatment options. Dietary supplements are considered an attractive alternative to traditional therapy, mainly because they are accessible to the general population. The aim of this study was to investigate the additive effects of a combination of energy restriction (ER) and four dietary supplements on changes in the anthropometric and biochemical parameters in 100 overweight or obese participants who were randomly assigned one of the dietary fibre supplements containing different dietary fibres or a placebo for 8 weeks. The results confirmed that fibre supplements plus ER significantly (*p* < 0.01) reduced the body weight, body mass index (BMI), fat mass, and visceral fat and ameliorated the lipid profile and inflammation at 4 and 8 weeks after the start of the study, while in the placebo group, significant differences in some parameters were observed only after 8 weeks of ER. A fibre supplement containing glucomannan, inulin, psyllium, and apple fibre was the most effective at reducing the BMI, body weight, and CRP (*p* = 0.018 for BMI and body weight and *p* = 0.034 for CRP compared to placebo at the end of the intervention). Overall, the results suggest that dietary fibre supplements in combination with ER may have additional effects on weight loss and the metabolic profile. Therefore, taking dietary fibre supplements may be a feasible approach to improve weight and metabolic health in obese and overweight individuals.

## 1. Introduction

Obesity has reached the epidemic level across the world, and its prevalence continues to increase [1]. Moreover, obesity is an important risk factor for various metabolic disorders, such as type 2 diabetes [2], hypertension, dyslipidaemia [3], cardiovascular disease [4], and others. Obese and overweight people have reduced wellbeing, an increased risk of mortality, and reduced life expectancy [5]. Because of the multifaceted nature of this problem, there is no single or simple approach to decrease the growing rate of obesity, and therefore new strategies are needed. Obesity and overweight are treated with a combination of caloric restriction and exercise (preferably aerobic) aimed at reducing fat accumulation and increasing energy expenditure [6]. However, diets and exercise sometimes fail to achieve their goal for various reasons: genetic background [7,8], low adherence to diet and exercise, or because patients tend to regain the lost weight in a short time after dieting [9]. Pharmacotherapy has emerged as an alternative or complementary therapy when lifestyle changes do not result in the desired weight loss [10]. Various medications with different mechanisms of action are available [11]. However, pharmacotherapy has some disadvantages, such as safety concerns or side effects.

Among the various alternatives for weight control, the use of dietary supplements from natural sources has become popular in recent years. A recent systematic review and meta-analysis of 62 human studies showed that viscous fibre intake affects body weight, body mass index (BMI), and waist circumference independent of an energy-restricted diet [12]. There is also extensive evidence that a high-fibre diet is associated with a lower risk of certain cancers, heart disease, diabetes, stroke, and all-cause mortality [13,14]. One proposed mechanism for the benefits of dietary fibre in weight management is appetite suppression and an increased feeling of satiety [15,16]. While fibre intake is generally efficient in this regard, the type of fibre must also be considered, as some types have minimal or negative effects on appetite, satiety, energy intake, and weight loss [17]. In addition, high-fibre foods are bulky and require more chewing, which likely leads to an improved feeling of satiety; thus, it cannot be directly inferred that the same type of fibre found in food is also effective in the form of a dietary supplement.

Common nondigestible carbohydrates (NDCs) in dietary supplements are glucomannan, inulin, psyllium, and β-glucans. Glucomannan is a natural dietary fibre composed of β 1,4-linked D-mannose and D-glucose monomers derived from a tuber called *Amorphophallus konjac* [18]. Its mechanism of action for weight loss may be based on its ability to absorb 50 times its weight in water volume, causing delayed gastric emptying along with a feeling of satiety [19]. Inulin is a soluble, nonviscous, fermentable dietary fibre. Some studies suggest that inulin may promote weight loss in obese, dyslipidaemic individuals with cardiovascular risk factors [20,21]. However, the most consistent finding in the literature is the ability of inulin to lower the concentration of triacylglycerols (TAGs) [22]. β-glucans are soluble, viscous, gel-forming dietary fibres, which increase the viscosity of the dietary pulp to slow nutrient absorption and improve glycaemic control and the lipid profile. In addition, beta-glucans are readily fermentable; fermentation results in the loss of the gel- and water-binding capacity [23]. Psyllium is a viscous, soluble, gel-forming, nonfermented fibre supplement. The advantage of taking psyllium compared to other soluble dietary fibres is that it is less easily fermented and therefore the prevalence of flatulence and abdominal bloating is fairly low [24]. Psyllium can affect body composition through several mechanisms, including gastric emptying [25], a feeling of satiety [26], the secretion of intestinal hormones, such as cholecystokinin [27], and by altering the glycaemic index or insulin response [28]. Different dietary fibres thus have variable effects on weight loss, and the health benefits of their consumption are also associated with positive effects on serum biochemical parameters. However, gastrointestinal symptoms, such as flatulence, bloating, constipation, and diarrhoea, may also accompany the increased fibre intake, the extent of which again largely depends on the type of fibre and its solubility and fermentability [29].

On the market, numerous dietary fibre supplements with different formulations are available. Because of the conflicting results regarding the nature of dietary fibre supplements for weight loss and the attenuation of certain obesity-related metabolic diseases, the objective of the present study was to investigate the influence of four commercially available dietary fibre supplements on the anthropometric and biochemical parameters in a population of overweight and obese patients who also had dyslipidaemia and mild hypertension. As energy restriction is the most accepted weight loss strategy, all supplementation interventions were performed in combination with energy restriction and compared to the energy restriction alone. Regardless of the putative beneficial health effects, the supplements will only be accepted by the consumer if they are also sensory-acceptable and do not cause gastrointestinal symptoms, and therefore the two parameters were followed concurrently. The hypothesis of our study is that dietary fibre supplements with energy restriction aid in weight loss and improve the lipid profile in overweight and obese participants.

## 2. Materials and Methods

### 2.1. Study Design

The study was a five-arm, parallel, randomized, control trial conducted at the University of Primorska, the Faculty of Health Sciences, between April and July 2022. The study protocol was approved by the Slovenian National Medical Ethics Committee (No. 0120-557/2017/4; Ministry of Health, Republic of Slovenia) and registered at ClinicalTrials.gov (NCT05333315). All subjects signed written informed consent forms before the conduction of the study and the Declaration of Helsinki was followed.

The participants were randomly allocated to one of five groups: the investigation product (IP1–IP4) groups or the placebo product (PP) group, before the initiation of an 8-week energy-restricted weight loss period. Stratified variables were gender and age. Blinding was not possible because some products were capsules, liquids, powder, or soft chews, and therefore the experiment was performed as an open-labelled experiment. The participants attended screening visits before the intervention period and on three clinical investigation days: at baseline (week 0), at the end of the first month of the intervention (week 4), and at the end of the whole intervention (week 8). At each time point, the participants had a consultation with a dietitian, including an evaluation of diet and body composition, blood pressure was measured, and blood samples were collected.

The primary outcome was the effect of dietary fibre supplements during energy restriction on body weight, compared with the placebo supplement. Secondary outcomes were the effects on body composition, blood pressure, gastrointestinal symptoms, and biochemical markers.

### 2.2. Study Participants

The study population included healthy adults (from 40 to 60 years old) recruited from the Primorska region of Slovenia. Volunteers were recruited through an advertisement posted on Internet forums, sent via e-mail lists, and published in local newspapers. Only healthy subjects were included. Additional inclusion criteria were a BMI between 25 and 30 kg/m^2^, no more than a 3% change in body mass within the last three months, age between 40 and 60 years, and a willingness to avoid the consumption of any food supplements at least 2 weeks before and during the study. The exclusion criteria included taking any prescribed medication or food supplement within the two weeks preceding the study; any clinically significant history of serious digestive tract, liver, kidney, cardiovascular, or haematological disease or diabetes; gastrointestinal disorders or other serious acute or chronic diseases. A study power calculation (power 0.9, alpha 0.05) was performed based on literature data [30,31]. We calculated that 20 subjects in each group would be sufficient to detect a 2-fold difference in the primary outcome between the groups taking the placebo and investigated supplements. To compensate for possible dropouts during the study, we decided to enlarge the study sample to 25 subjects. A total of 145 participants were recruited into the study. A total of 45 participants did not meet the inclusion criteria and therefore the final analytical sample was 100 participants.

Once eligibility was confirmed, the following information was collected: age, gender, and ethnic origin of the subject; height (cm) and body weight (kg); body fat mass (kg); and visceral fat index. The participants did not receive any compensation for their participation in the study.

### 2.3. Intervention

The study was conducted with four different dietary fibre supplements (IP formulations) and a placebo product (PP) produced by Biostile d. o. o., Comen, Slovenia. The details on the content, form, and administration of the different dietary fibre supplements are shown in Table 1. Briefly, IP1 in soft-chew form contained only glucomannan; IP2 in powder form contained mainly psyllium, glucomannan, inulin, and apple fibre; IP3 in powder form contained mainly psyllium, inulin, and apple fibre, but also other plant extracts; IP4 in liquid form contained arabinogalactan, inulin, and beta-glucans; and PP in capsule form contained maltodextrin. Participants were informed of the importance of consuming water with the supplements.

At the beginning of the study, an energy-restriction plan was prepared for each participant. After nutritional data collection at baseline, all subjects participated in two educational sessions (2 h) on healthy diet, nutrient composition, the proper timing of eating, and the beneficial effects of the daily consumption of vegetables and fruits. All subjects also attended two sessions of individual consultations, during which they were given a personalized diet plan for eight weeks. To estimate the total energy needs, an individual’s RMR measured with an indirect calorimeter (MedGem^®^ Microlife, Medical Home Solutions, Inc., Golden, CO, USA) was multiplied by the appropriate factor of physical activity (from 1.3 to 1.6), and then a reduction of 837 kJ/200 kcal–2093 kJ/500 kcal was made. The macronutrient composition of the diet was approximately 48–52% EI of carbohydrates, 30–33% EI of fat, and 18–22% EI of protein. Dietary intake of the participants was tracked during the study using a 24 h recall. An expert dietitian carefully collected a list of all foods and beverages, using food models. Dietary data were analysed using the Open Platform for Clinical Nutrition (OPEN), which can be accessed through the website http://opkp.si/ (accessed on 2 October 2022).

Adherence to the supplementation protocol was monitored by a researcher who contacted subjects once a week. Each subject was also required to return the original bottle of their respective supplement.

### 2.4. Anthropometric Measurements

All measurements were performed following an overnight fast between 7 a.m. and 8 a.m. in standardised conditions by the same examiner. Participants voided their bladders before the anthropometric measurements were performed. Body weight was measured in light clothing without shoes, to the nearest 0.1 kg, and height to the nearest 0.1 cm, using a Leicester Height Measure (Invicta Plastics Limited, Oadby, UK). BMI was calculated as weight (kg) divided by height (m) squared. Body composition (total percentage body fat and fat-free mass (FFM)) was assessed using bioelectrical impedance analysis (BIA) Tanita MC-980MA (Maeno-cho, Japan) and dedicated software (GMON Pro-Tanita).

Additionally, blood pressure was measured with an automatic device (automatic blood pressure monitor, Model SEM-1, Omron Healthcare Company, Singapore) after 10 min of rest in the supine position. The measurement was repeated twice.

### 2.5. Biochemical Analysis

Venous blood samples were collected in 6 mL vacuum test tubes (Beckton, Dickinson and company, Franklin Lakes, NJ, USA) after a 12 h overnight fast (complete dietary restriction with the exception of water). Serum was immediately separated by a 10 min centrifugation at 2000× *g*, frozen, and stored at −80 °C until subsequent analysis. Serum concentrations of glucose, TAGs, total cholesterol, LDL cholesterol, HDL cholesterol, total bilirubin, AST, ALT, total bilirubin, uric acid, and C-reactive protein (CRP) were measured with a Cobass c111 analyser (Roche). Serum concentrations of interleukin-6 (IL-6), tumour necrosis factor alpha (TNF-α), lipopolysaccharide binding protein (LBP), and zonulin were determined in duplicate on a microplate reader (Tecan, Männedorf, Switzerland) using human ELISA Kits (BioVendor, Brno, Czech Republic for IL-6, TNF-α, and LBP, and MyBiosource, San Diego, CA USA, for zonulin). Assay sensitivities were 0.32 pg/mL for IL-6, 0.13 pg/mL for TNF-α, 0.13 ng/mL for LBP, and 0.5 ng/mL for zonulin. Assay interassay and intraassay CVs were typically between 5% and 12%.

### 2.6. Product Evaluation, Changes in Appetite and Adverse Events

During the fourth week of the intervention, the participants were asked to evaluate the products. The participants were provided a prescribed questionnaire that contained three items: the overall impression and likeability of the product, and the acceptability of its taste and its texture. The evaluation scores were from 1 (dislike very much) to 10 (like very much) for the likeability of the product, and from 1 (dislike very much) to 5 (like very much) for taste and texture, separately.

Moreover, participants were also asked to report how likely they were to continue consuming the product, to report the possible gastrointestinal discomforts, and to self-report changes in appetite while consuming the product. Gastrointestinal discomforts, self-reported changes in appetite, concomitant medication, and adverse events were registered on the clinical investigation days and during the consultations with the dieticians.

### 2.7. Statistical Analysis

Statistical analysis was performed using SPSS version 23.0 (IBM Corp., Armonk, NY, USA). The statistical analysis was performed in two ways: an intention-to-treat analysis was performed on age, body weight, BMI, and fat mass, in which all participants were included, and a per protocol analysis of the treatment efficacy, which was performed on all participants who completed the 8-week intervention. All results are presented as the mean value with standard deviation (SD). Normality of variables was tested using the Shapiro–Wilk test. The effects of the interventions within each group were analysed with a Student’s paired-samples *t*-test or Wilcoxon signed-rank test, whereas the comparison of the mean changes between groups was analysed using an independent *t*-test or the Mann–Whitney U test. Moreover, to compare the means at baseline or the change at week 4 or 8 weeks from baseline between the groups, the one-way ANOVA was performed, and *p*-values < 0.05 were considered statistically significant.

## 3. Results

### 3.1. Baseline Characteristics of Participants

**Figure 1:**
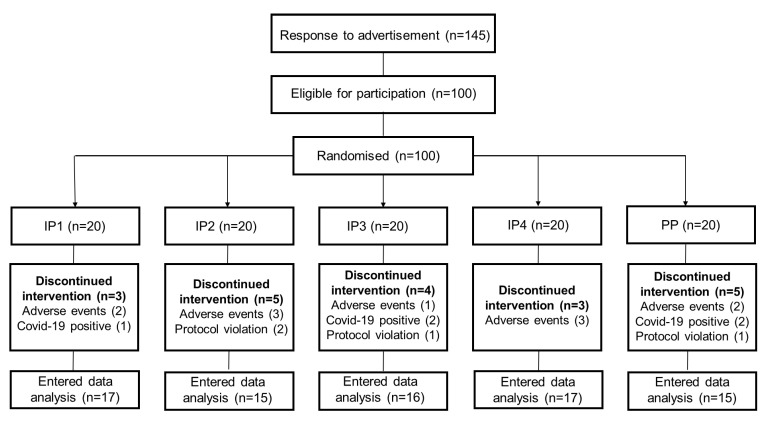
Flowchart.

Overall, 100 participants were included in the study. There were 83 females and 17 males, evenly distributed across the five treatment groups. In total, 80 of the 100 participants (20% dropout) completed the 8-week study. Six participants were COVID-19-positive during the study (IP1, *n* = 1; IP3, *n* = 2; IP4, *n* = 1; PP, *n* = 2), ten participants withdrew due to adverse symptoms (stomach upsets, *n* = 4; diarrhoea, *n* = 3; difficulty in swallowing recommended dose of IP2/IP3, *n* = 3), and four participants were excluded because of protocol violation (IP2, *n* = 2; IP3, *n* = 1; PP, *n* = 1). The flowchart of the study is illustrated in Figure 1.

Baseline data (Table 2) show the characteristics of the study participants (*n* = 100) randomised to five groups. At baseline, there were no significant differences (*p* > 0.05) in age, body weight, BMI, or fat mass across the five groups. If we consider only the participants who completed the study, then the BMI was significantly lower in the PP group. However, the body weight, percentages of fat mass and fat-free mass, and visceral fat rating did not significantly differ between the groups (Table 3).

### 3.2. Adherence

Adherence to the treatment (monitored by the investigator by counting the medication bags returned by patients at each follow-up visit) and adherence to diet (assessed by patients at each follow-up visit) were generally high in all groups (>85% at all follow-up visits). No statistically significant differences were found between the groups in the adherence or the self-assessment of the level of adherence to the diet. Overall, the self-reported compliance was 89% consumption of the intended intervention products, with 92% in the IP1 group, 87% in the IP2 group, 86% in the IP3 group, 93% in the IP4 group, and 90% in the PP group.

### 3.3. Effects of Fibre Supplementation during Energy-Restricted Diet on Anthropometric Parameters

The first objective was to test whether dietary fibre supplements combined with energy restriction are able to reduce body weight and BMI. The result confirmed that the administration of IP1, IP2, IP3, and IP4 significantly (*p* < 0.01) reduced body weight and BMI 4 and 8 weeks after the start of the study, while in the PP group, the significant differences in body weight and BMI were observed only after 8 weeks of energy restriction (Table 3). The study found that the reduction in body weight and BMI was significantly different between the IP2 and PP (*p* = 0.002 for BMI and body weight), IP3 and PP (*p* = 0.050 for body weight and *p* = 0.029 for BMI), and IP4 and PP (*p* = 0.027 for body weight and *p* = 0.028 for BMI) groups after 4 weeks of intervention. Moreover, the decrease after 4 weeks of supplementation was significantly higher in the IP2 group than in the IP1 group (*p* = 0.041 for body weight and *p* = 0.021 for BMI) and IP3 group (*p* = 0.048 for body weight). After 8 weeks of the intervention, reductions in body weight and BMI were significantly different only between PP and IP2 (*p* = 0.018 for BMI and body weight) and between PP and IP4 for BMI (*p* = 0.049).

**Figure 2:**
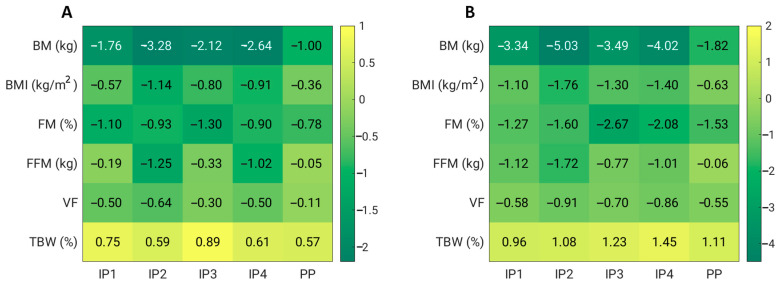
Effects of dietary fibre supplementation on anthropometric parameters after 4 weeks (**A**) and 8 weeks (**B**). BM—body mass; BMI—body mass index; FM—fat mass; FFM—fat-free mass; TBW—total body water; VF—visceral fat.

In addition, we also analysed whether the visceral mass and fat mass were reduced as a function of weight loss (Table 3, Figure 2). Indeed, both fat mass and visceral fat were significantly reduced after 4 weeks (*p* < 0.01) in groups IP1, IP2, IP3, and IP4, and this reduction was even greater after 8 weeks (*p* < 0.01) compared to the baseline measurement. However, the reduction in fat mass and visceral fat was also significant in the PP group after 8 weeks of energy restriction. The study showed that the reduction in visceral fat was significantly different only between PP and IP2 (*p* = 0.019), but only after 4 weeks of the intervention. However, the FFM was also significantly affected in all groups (except PP). A significantly higher reduction was observed in the IP2 group than in the PP group (*p* = 0.021) and in the IP1 group than in the PP group (*p* = 0.032) after 4 weeks of intervention, and even after 8 weeks of intervention (IP2 vs. PP, *p* = 0.008, and IP1 vs. PP, *p* = 0.007). Overall, the IP2 supplementation was the most effective at reducing not only body weight, BMI, and visceral fat, but also FFM (Figure 2).

**Figure 3:**
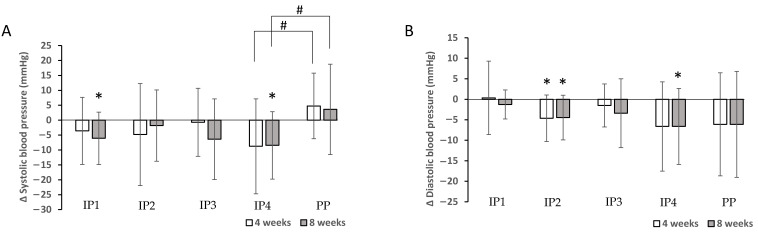
Effects of dietary fibre supplementation on systolic blood pressure (SBP) (**A**) and diastolic blood pressure (DBP) (B) after 4 and 8 weeks. * Significant decrease in SBP and DBP compared with the baseline measurement (*p* < 0.050). # Significantly different decrease between intervention and placebo group (*p* < 0.050).

In addition, SBP decreased significantly after 8 weeks of the intervention (*p* < 0.01) in groups IP1 and IP4, while DBP decreased significantly in groups IP2 and IP4 after 4 weeks (IP2) and 8 weeks (IP2 and IP4) compared with the baseline measurement (Table 3, Figure 3). The study found that the decrease in SBP was significantly different between the IP4 and PP groups (*p* = 0.049 at 4 weeks and *p* = 0.050 at the end of the intervention). No other differences were found in relation to blood pressure (Figure 3). Overall, a significant effect on blood pressure (SBP) was observed only in the IP4 group compared to the placebo group.

### 3.4. Effects of Fibre Supplementation during Energy-Restricted Diet on Metabolic Outcomes

**Figure 4:**
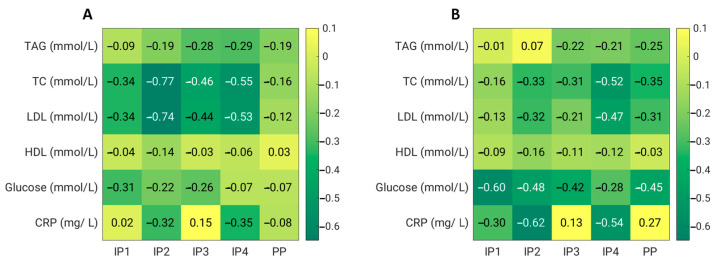
Effects of dietary fibre supplementation on biochemical parameters after 4 weeks (**A**) and 8 weeks (**B**). CRP—C-reactive protein; HDL—high-density lipoprotein cholesterol; LDL—low-density lipoprotein cholesterol; TAG—triacylglycerol; TC—total cholesterol.

Fibre supplements combined with energy restriction were able to significantly lower the total, HDL, and LDL cholesterol in the IP2 and IP4 groups 4 weeks and 8 weeks after the start of the study. The serum total and LDL cholesterol decreased significantly more in the IP2 group compared to the placebo group at week 4 (*p* = 0.020 for total cholesterol and *p* = 0.016 for LDL cholesterol); at week 8, the differences were no longer significant (Table 4, Figure 4). In the IP3 group, only a transient but significant decrease in the total and LDL cholesterol was observed after 4 weeks of the intervention.

For plasma glucose, we did not observe any significant effect of dietary fibre supplementation compared with the placebo. However, in the IP1, IP2, IP3, and PP groups, the serum glucose significantly decreased at week 8 compared with the baseline values. In addition, we did not detect any significant treatment effect on the serum levels of triacylglycerols compared with the placebo. However, in the IP3 and IP4 groups, the TAGs significantly decreased at week 4 compared with the baseline levels (Table 4, Figure 4).

### 3.5. Effects of Fibre Supplementation during Energy-Restricted Diet on Inflammatory Parameters and Gut Permeability

**Figure 5:**
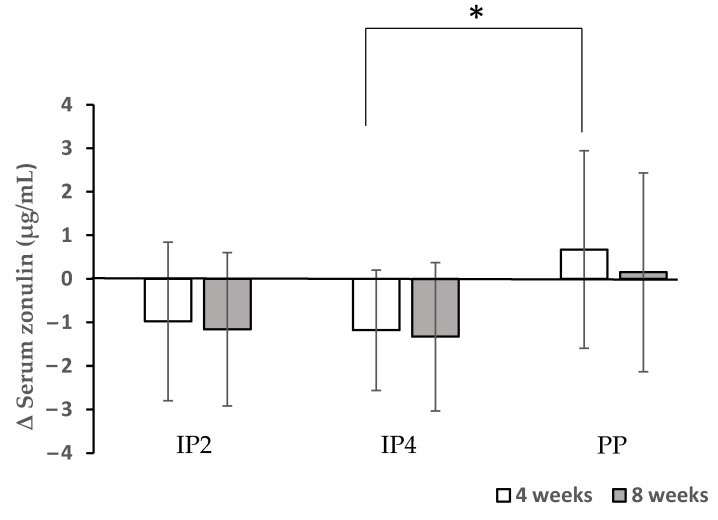
Effects of dietary fibre supplementation on serum zonulin levels. * The reduction in the zonulin levels was significantly different between the IP4 and PP group at week 4 (*p* = 0.046).

Dietary fibre supplements in the IP1, IP2, and IP4 groups combined with energy restriction were able to significantly decrease the total CRP 8 weeks after the start of the study. The CRP levels decreased significantly more in the IP2 group compared to the placebo group at week 8 (*p* = 0.034) (Table 5). The serum levels of IL-6 and TNF-alpha were not significantly affected by either the fibre supplementation or energy restriction. In addition, the serum levels of LBP decreased significantly after a 4-week intervention compared with the baseline levels when a high-fibre diet was combined with energy restriction in the IP4 and IP2 groups (Table 5). Due to the promising effects of IP2 and IP4 for a potential reduction in the inflammatory status, the zonulin levels were measured only in these two groups in comparison to the PP group. Zonulin decreased significantly after a 4-week intervention compared with the baseline levels when a high-fibre diet was combined with energy restriction in the IP4 group (Figure 5). The reduction in the zonulin levels was significantly different between the IP4 and PP group at week 4 (*p* = 0.046) (Figure 5).

### 3.6. Product Evaluation

Participants were asked to rate how much they liked the product in general, and how acceptable it was in terms of taste and texture (Table 6). The mean scores for the likeability were 4.8 out of 10 for IP2, 6.3 for PP, 6.8 for IP3, 7.1 for IP1, and 7.3 for IP4. Additionally, the mean scores for taste acceptability were 4.0, 3.9, 3.9, and 3.7 out of 5 for PP, IP4, IP1, and IP3, respectively, whereas the mean score for IP2 was 2.9 out of 5. Moreover, the mean score for texture acceptability was the lowest in IP2 (1.8 out of 5) and the highest in IP4 (4.3 out of 5), while for all the other groups, the scores were 3.1 for IP3, 3.8 for PP, and 3.9 for IP1. Some participants in the IP2 group described the product as “grainy” and “unpalatable” and had difficulties swallowing it. A total of 60% of the participants were not likely to consume IP2, 50% of the participants were not likely to consume IP1 and IP4, 36% of the participants were not likely to consume IP3, and 12.5% of participants were not likely to consume PP after the end of the study.

As shown in Table 7, the participants reported some adverse or side effects of the fibre supplement consumption. The three main complaints were flatulence, abdominal discomfort (feeling too full), and changed bowel habits (constipation), and they were rated as mild or moderate. The participants receiving the fibre supplement IP2 experienced a higher degree of gastrointestinal discomfort and flatulence (55%) in comparison to the other groups (0–20%). Participants from all experimental groups reported feeling too full following the consumption of the product (15% in IP1, 13% in IP2, 17% in IP3, and 7% in IP4), whereas in the PP group, no side effects were reported. Moreover, in all experimental groups, except in the placebo group, participants reported decreased appetite (15% in IP1, 13% in IP2, 17% in IP3, and 7% in IP4).

### 3.7. Dietary Intake

Because energy restriction was prescribed to the participants of all groups, dietary intakes were analysed for the baseline and for week 8. Even though the energy intake decreased in all the groups and the macronutrient composition of the diets changed due to the dietary recommendations, neither the energy intake nor the macronutrient composition of the dietary intake differed between the five groups (Table 8). This also applied to the dietary fibre content when the fibre content of the treatment products was not included. In general, in all the experimental groups, the participants did not reach the recommendation for fibre intake, which is 30 g of dietary fibre per day [32].

## 4. Discussion

Many studies have found that increasing fibre intake results in increased satiety, reduced hunger, decreased energy intake, and weight loss [33,34]. The present results support the hypothesis that dietary fibre supplements in combination with conventional energy restriction may have additional effects on weight loss and the metabolic profile. However, the effects depend on the product formulation.

The influence of dietary fibre supplements on energy regulation remains controversial [15]. Therefore, the main objective of the present study was to compare the weight-reducing effects of different commercial dietary fibre supplements vs. a placebo in combination with energy restriction in overweight and obese subjects. The commercial product formulations varied largely among themselves—in composition, the amounts of active substances present, and form. The fibre supplements used were glucomannan (IP1), a combination of glucomannan, inulin, psyllium, and apple fibre (IP2), a combination of inulin, psyllium, apple fibre, and various extracts (IP3), and a combination of inulin, β-glucans, and arabinogalactan (IP4). Our results show that taking 3 g of glucomannan per day alone (IP1) did not result in a significant decrease in body weight or BMI compared with the placebo at 4 and 8 weeks. The results are consistent with a meta-analysis that found a nonsignificant decrease in body weight after glucomannan supplementation [35]. However, the present results are inconsistent with a previous systematic review that found a statistically significant decrease in body weight [36] after glucomannan supplementation and do not support the recommendation of the European Food Safety Authority for the use of glucomannan as a weight loss aid in combination with an energy-restricted diet [37]. However, when glucomannan (4.3 g/day) was used in combination with inulin (2.5 g/day), psyllium (10 g/day), and apple fibre, a significant decrease in body weight and BMI was observed compared with the placebo. Recently, a systematic review and meta-analysis showed no significant effect of psyllium on body weight, body mass index, and waist circumference compared with the control group in adults [38]. Similarly, it was reported that inulin supplementation did not result in additional weight loss when used concurrently with moderate energy restriction [20]. In our case, the combination of inulin (nonviscous fibre), glucomannan (viscous fibre), and psyllium (less fermentable fibre) in IP2 was not only significantly more effective than glucomannan alone in IP1, but also more than IP3, where inulin, apple fibre, and psyllium were mixed. Thus, our study showed that the right combination of mixed dietary fibres is crucial to observe specific effects on weight loss and BMI.

In addition, a similar combination (inulin (nonviscous fibre) and β-glucans (viscous fibre)) in the IP4 group also significantly reduced body weight and BMI at 4 weeks and BMI at 8 weeks in comparison to the placebo. These results are very interesting, as the amount of fibre in this supplement was very low compared to the literature; nonetheless, this product showed very good results. It has been reported that the ingestion of β-glucans in an amount ≥ 3 g causes a decrease in body weight in people with type 2 diabetes for at least 3 weeks [39,40]. The viscosity of soluble dietary fibre plays an important role in appetite control and satiety. High-viscosity β-glucans delay gastric emptying and slow down digestion and the absorption of nutrients due to the lesser effect on enzymatic activity and mucosal absorption, cause an earlier feeling of satiety, and reduce energy intake. In addition, short-chain fatty acids produced during β-glucan fermentation regulate the release of various gastrointestinal hormones that play an important role in signalling the feeling of satiety. Another mechanism by which soluble dietary fibre, including β-glucans, induces the feeling of satiety is by reducing glycaemic and insulinemic responses [41,42].

Overall, IP2 and IP4 were the most effective supplements in reducing body weight (−5 kg for IP2 and −4 kg for IP4 in 8 weeks) and BMI. It is known that even slight reductions in weight can produce metabolic improvements. Each kilogram of lost body weight has been connected with a 16% reduction in type 2 diabetes risk [43]. Nevertheless, we observed stronger effects of the fibre supplements on the anthropometric parameters after 4 weeks, and these effects were quite similar between the experimental products (IP2, IP3, IP4). However, these effects were weaker (IP2 and IP4) or not present after 8 weeks (IP3), indicating that fibre supplements might have time-limited effects.

The present study and others [20] report that weight loss due to energy restriction is effective at lowering blood pressure. The present study suggests that supplementation with IP4 has an additional blood-pressure-lowering effect during energy restriction. It has been reported that β-glucans (present in IP4) can reduce blood pressure to normal levels [44]. Indeed, in the present study, only the IP4 participants had significantly reduced SBP at 4 and 8 weeks after the intervention compared to the placebo group. Consistent with our findings, although differing in the amount, material source, and physiochemical properties of β-glucans, a study of hypertensive and hyperinsulinaemic individuals showed significant reductions in systolic and diastolic blood pressure in the group consuming oats (standardised to 5.52 g/day β-glucans) for 6 weeks compared with the group consuming low-fibre grains (total fibre < 1 g/day) [45]. Moreover, this effect could be mediated in part by the fibre-induced changes in the gut microbiota, as it is known that microbial metabolites and other byproducts (e.g., SCFAs, trimethylamine-N-oxide, and lipopolysaccharides) could act on downstream cellular targets involving the kidney, endothelium, and heart, and help prevent or exacerbate the development of hypertension [20,46]. Consuming a fibre supplement and having an energy-restricted diet both reduced the serum total cholesterol and LDL levels. In our study, the four groups receiving the dietary fibre tended towards greater improvement in the lipid profile than did the control individuals at 4 weeks. However, the lack of significant differences in the total cholesterol, LDL cholesterol, and TAGs observed between the groups at 8 weeks does not support the hypothesis that this type of fibre has any effect on the lipid profile after a longer period of the intervention. Whether significant differences would be observed by increasing the fibre dose is worth considering in future studies. The greatest impact on lowering the total and LDL cholesterol was IP4, for which the doses were much smaller than reported in the literature for similar fibre formulations [47]. The fibres in our IP3 formulation were very well dispersed, as IP4 was provided in the form of a solution. The physicochemical properties of fibre supplements are very important and contribute to the effects on serum cholesterol [47]. However, there were significant differences in the cholesterol levels between the IP4 and placebo groups only at 4 weeks of the intervention, indicating the short-term effects of fibre supplementation and/or the crucial role of an energy-reduced diet. Many clinical and animal trials have demonstrated the hypocholesterolaemic effects of soluble fibres [48,49,50]. In a meta-analysis evaluating the results of 30 studies, the ingestion of β-glucans (also found in IP4) significantly lowered the total cholesterol, LDL cholesterol, and TAG levels [51]. Glucans have been reported to decrease the reabsorption of bile acids and increase transport to the colon due to high viscosity, activate 7α-hydroxylase to cause the excretion of cholesterol from the body, and increase the upregulation of the low-density lipoprotein receptor (LDLR), allowing the transport of LDL to hepatocytes and the conversion of cholesterol to bile acids [52,53]. Another cholesterol-lowering effect of β-glucans is their fermentation ability [53]. However, we must point out that our participants in the IP4 study consumed 30 mg of beta-glucans per day, whereas the Food and Drug Administration (FDA) recommends the consumption of at least 0.75 g per serving to achieve such health benefits [54]. Nevertheless, studies have shown that for every 1 mg/dL reduction in a patient’s LDL cholesterol concentration, the relative risk of developing coronary heart disease decreases by 1%. Therefore, the within-group reduction in the LDL cholesterol levels observed in our study is not only statistically significant but is also likely to be clinically important [55]. The present study reports that a supplement of various types of fibre does not provide additional effects on the glucose levels when applied concomitantly with a moderate dietary-energy-restriction regimen.

There is no consensus in the literature on the anti-inflammatory effects of dietary fibre. The results of two epidemiological studies suggest that the consumption of dietary fibre is inversely associated with serum CRP concentrations [56,57], but another large study failed to find any association between the consumption of whole-grain products and plasma CRP, IL -6, or fibrinogen concentrations. [58]. Moreover, recent studies have shown that fermentable fibre can modulate systemic inflammatory markers in mice [59] and men [60]. Indeed, the daily intake of 10 g (mainly fermentable) dietary fibre in haemodialysis patients decreased blood inflammatory markers, such as TNF-α and CRP [60]. Additionally, it was suggested that 2% (*w*/*w*) of fibre treatment (with glucomannan, inulin, or both) beneficially enhanced the mucosal barrier function and anti-inflammatory status in mice [59]. Our results showed that only the combination of glucomannan, psyllium, and inulin (IP2) had an additional effect on the serum CRP levels compared to the placebo, while IP4 (the combination of inulin and β-glucans) significantly reduced the zonulin levels in comparison to the placebo and therefore improved the mucosal barrier function.

In this study, we also investigated whether 8 weeks of increased fibre intake would influence the participants’ gastrointestinal comfort, whether the increased fibre intake was acceptable for the participants, and whether the consumption of dietary fibre supplements led to decreased appetite. After 4 weeks of dietary fibre supplementation, the data indicated an increase in the frequency of bloating and a hardening of the stool consistency in all four conditions of dietary fibre supplementation. There was no evidence that other side effects, such as bloating or cramping, increased under fibre supplementation. This suggests that, on the whole, the participants did not experience significant adverse gastrointestinal reactions due to the increased fibre intake. However, there was some abdominal discomfort and flatulence, which is in line with previous studies that report the positive associations between fibre intake and increased flatulence, bloating, uncomfortable abdominal distention, and changed stool consistency [61,62]. Moreover, fibre supplements have been shown to reduce appetite and increase feelings of satiety and therefore help with weight loss [15,16]. However, in our study, in all the experimental groups, only 10–20% of the participants reported decreased appetite and feeling too full during supplement consumption. Although IP2 and IP4 were the most effective supplements for weight loss and lowering the total and LDL cholesterol, 60% of the participants were not likely to consume IP2 and 50% of the participants were not likely to consume IP4 after the end of the study.

The results of this study were limited by a relatively small sample size, which may explain the lack of significant results when comparing between groups. In addition, meals and the consumption of the test products were self-administered; thus, the possibility of noncompliance could not be excluded. Misreporting of intake may have occurred in the current study.

## 5. Conclusions

In conclusion, based on the improvements in the health parameters observed in the present study, and because of the extensive dietary fibre intake gap between the recommended intake and actual dietary intake in Slovenia [63], fibre supplementation may be a potential approach to improve weight and metabolic health in obese and overweight individuals, but it should not replace a balanced diet with fibre-rich foods.

## Figures and Tables

**Table 1:** Dietary fibre supplements (IP1–IP4) and placebo product (PP): overview.

Company Product Name	Form	Administration	Ingredients—In Total
Product BSMS (IP1)	Soft chew	6 soft chews/day (with water): 2 soft chews 30 min before main meal (breakfast, lunch, dinner).	3 g glucomannan
Product BVCF (IP2)	Powder	1 bag/day (with water) 30 min before breakfast.	4.3 g glucomannan 7 g psyllium 2.5 g inulin 1 g apple fibre
Set BPRX (IP3)	Powder capsule	1 bag/day (with water) 30 min before breakfast. 3 capsules/day (with water or tea): one at breakfast, one at lunch, and one at dinner.	10 g psyllium 2 g inulin 1.5 g apple fibre 500 mg apple pectin seed extracts (pumpkin (300 mg), cumin (150 mg), anise (150 mg)), leaf extracts (artichoke (150 mg), peppermint (150 mg)), root of pellitory (500 mg), garlic extract odourless (300 mg), curcuma rhizome extract (150 mg)
Product BLMB (IP4)	Liquid	2 × 20 mL/day (diluted in water) 30 min before breakfast and 30 min.	700 mg inulin 700 mg arabinogalactan 30 mg β-glucans
Placebo product (PP)	Capsule	3 capsules/day (with water): one at breakfast, one at lunch, and one at dinner.	420 mg maltodextrin

IP: investigation product; PP: placebo product.

**Table 2:** Baseline characteristics of all included participants.

	IP1	IP2	IP3	IP4	PP	*p*
N (F/M)	20 (16/4)	20 (17/3)	20 (17/3)	20 (16/4)	20 (17/3)	
Age (years)	48.8 ± 8.6	50.1 ± 5.1	51.5 ± 6.7	46.3 ± 5.3	50.7 ± 6.0	0.109
Body weight (kg)	86.9 ± 10.7	87.7 ± 16.9	82.0 ± 8.8	85.9 ± 15.1	77.4 ± 8.1	0.122
BMI (kg/m^2^)	29.1 ± 3.5	30.0 ± 4.3	29.8 ± 2.9	29.4 ± 4.2	28.0 ± 2.2	0.095
Fat mass (%)	33.8 ± 8.2	37.4 ± 5.7	35.4 ± 5.1	30.9 ± 7.5	30.0 ± 8.1	0.080

*p*-value denotes differences in means between different groups using one-way ANOVA test.

**Table 3:** Effects of fibre supplementation during energy-restricted diet on anthropometric parameters.

	IP1 Mean ± SD	IP2 Mean ± SD	IP3 Mean ± SD	IP4 Mean ± SD	PP Mean ± SD	*p*
N (F/M)	17 (14/3)	15 (14/1)	16 (15/1)	17 (15/2)	15 (14/1)	
**ANTHROPOMETRIC** **PARAMETERS**						
**Body weight (kg)**						
Baseline	87.5 ± 11.4	91.8 ± 18.5	82.3 ± 9.2	84.9 ± 14.8	76.9 ± 9.8	0.160
After 4 weeks	85.8 ± 11.5 ^b^	88.5 ± 17.8 ^b^	80.2 ± 8.9 ^b^	81.9 ± 13.7 ^b^	75.9 ± 8.9	0.019 ^c^
After 8 weeks	84.2 ± 11.1 ^b^	86.7 ± 18.4 ^b^	78.8 ± 9.0 ^b^	80.5 ± 13.7 ^b^	74.4 ± 7.8 ^b^	0.054
**BMI (kg/m^2^)**						
Baseline	29.3 ± 3.7	31.7 ± 3.9	30.1 ± 2.9	29.4 ± 4.2	27.2 ± 2.8	0.029 ^a^
After 4 weeks	28.7 ± 3.8 ^b^	30.5 ± 3.7 ^b^	29.3 ± 2.8 ^b^	28.4 ± 4.0 ^b^	25.8 ± 2.4	0.012 ^c^
After 8 weeks	28.2 ± 3.6 ^b^	29.9 ± 3.9 ^b^	28.8 ± 2.8 ^b^	27.9 ± 4.0 ^b^	25.3 ± 2.4 ^b^	0.044 ^c^
**Fat mass (%)**						
Baseline	33.8 ± 8.2	38.5 ± 5.7	36.1 ± 5.3	31.1 ± 7.7	30.6 ± 5.7	0.073
After 4 weeks	32.0 ± 8.1 ^b^	37.6 ± 6.1 ^b^	34.8 ± 5.4 ^b^	30.2 ± 7.8 ^b^	29.8 ± 8.9	0.804
After 8 weeks	31.8 ± 8.6 ^b^	36.9 ± 5.6 ^b^	33.4 ± 5.4 ^b^	29.0 ± 7.4 ^b^	29.4 ± 8.5 ^b^	0.190
**Fat-free mass (kg)**						
Baseline	58.4 ± 10.2	56.0 ± 11.3	52.4 ± 5.8	57.9 ± 10.2	53.3 ± 9.3	0.508
After 4 weeks	58.2 ± 10.1	54.8 ± 10.8 ^b^	52.1 ± 5.5	56.9 ± 9.9 ^b^	53.2 ± 9.1	0.032 ^c^
After 8 weeks	57.3 ± 10.2 ^b^	54.3 ± 11.4 ^b^	51.7 ± 5.2 ^b^	56.9 ± 9.7 ^b^	52.5 ± 8.2	0.025 ^c^
**Visceral fat rating**						
Baseline	9.5 ± 3.3	9.7 ± 3.3	9.1 ± 2.4	8.1 ± 2.5	7.2 ± 1.8	0.232
After 4 weeks	9.0 ± 3.2 ^b^	9.1 ± 3.2 ^b^	8.8 ± 2.2	7.6 ± 2.3 ^b^	7.1 ± 1.8	0.209
After 8 weeks	8.9 ± 3.4 ^b^	8.8 ± 3.3 ^b^	8.4 ± 2.4 ^b^	7.3 ± 2.3 ^b^	6.5 ± 1.9 ^b^	0.583
**Total body water (%)**						
Baseline	47 ± 6	43 ± 4	45 ± 4	49 ± 5	49 ± 6	0.079
After 4 weeks	48 ± 6 ^b^	44 ± 4 ^b^	46 ± 4 ^b^	50 ± 5 ^b^	50 ± 6 ^b^	0.842
After 8 weeks	48 ± 6 ^b^	44 ± 4 ^b^	46 ± 4 ^b^	50 ± 5 ^b^	50 ± 6 ^b^	0.722
**BLOOD PRESSURE**						
**Systolic blood pressure**						
Baseline	129 ± 9	128 ± 14	127 ± 14	129 ± 17	121 ± 24	0.831
After 4 weeks	125 ± 14	124 ± 14	126 ± 12	120 ± 11	126 ± 15	0.280
After 8 weeks	123 ± 11 ^b^	126 ± 15	120 ± 17	120 ± 14 ^b^	121 ± 14	0.217
**Diastolic blood pressure**						
Baseline	81 ± 6	83 ± 5	81 ± 7	83 ± 7	82 ± 15	0.948
After 4 weeks	81 ± 10	78 ± 6 ^b^	80 ± 6	76 ± 8	76 ± 8	0.297
After 8 weeks	80 ± 5	78 ± 5 ^b^	78 ± 8	76 ± 7 ^b^	74 ± 11	0.559

ANOVA was performed to compare the means at baseline or the change at week 4 or 8 weeks from baseline between the groups. *p* ^a^-value denotes differences in means between different groups using one-way ANOVA test; *p* ^b^-value denotes significant (*p* < 0.05) difference from the initial value within group determined by a Student’s paired-samples *t*-test or Wilcoxon signed-rank test; *p* ^c^-value denotes differences in the change at week 4 or 8 weeks from baseline between the groups determined by one-way ANOVA.

**Table 4:** Effects of fibre supplementation during energy-restricted diet on metabolic parameters.

	IP1 Mean ± SD	IP2 Mean ± SD	IP3 Mean ± SD	IP4 Mean ± SD	PP Mean ± SD	*p*
N (F/M)	17 (14/3)	15 (14/1)	16 (15/1)	17 (15/2)	15 (14/1)	
**BIOCHEMICAL** **PARAMETERS**						
**Triacylglycerols (mmol/L)**						
Reference range	0.6–1.7 mmol/L					
Baseline	1.06 ± 0.61	1.52 ± 0.54	1.39 ± 0.34	1.39 ± 0.42	1.48 ± 0.79	0.316
After 4 weeks	0.97 ± 0.40	1.32 ± 0.47	1.12 ± 0.29 ^a^	1.10 ± 0.28 ^a^	1.29 ± 0.59	0.878
After 8 weeks	1.05 ± 0.53	1.58 ± 0.73	1.17 ± 0.38	1.18 ± 0.49	1.21 ± 0.32	0.596
**Total cholesterol (mmol/L)**						
Reference range	4.0–5.2 mmol/L					
Baseline	4.93 ± 1.16	5.56 ± 0.51	5.32 ± 0.46	5.74 ± 1.28	5.82 ± 1.31	0.247
After 4 weeks	4.59 ± 1.05	4.80 ± 0.44 ^a^	4.86 ± 0.77 ^a^	5.19 ± 1.26 ^a^	5.67 ± 0.99	0.165
After 8 weeks	4.77 ± 1.21	5.23 ± 0.59 ^a^	5.01 ± 0.33	5.22 ± 1.10 ^a^	5.35 ± 0.91	0.479
**LDL cholesterol (mmol/L)**						
Reference range	2.0–3.3 mmol/L					
Baseline	3.79 ± 1.21	4.58 ± 0.60	4.14 ± 0.46	4.66 ± 1.47	4.53 ± 1.55	0.337
After 4 weeks	3.45 ± 1.21	3.83 ± 0.59 ^a^	3.70 ± 0.58 ^a^	4.13 ± 1.45 ^a^	4.41 ± 1.17	0.157
After 8 weeks	3.66 ± 1.32	4.25 ± 0.68 ^a^	3.92 ± 0.45	4.18 ± 1.25 ^a^	4.11 ± 1.19	0.508
**HDL cholesterol (mmol/L)**						
Reference range	>1.4 mmol/L (M) >1.6 mmol/L (F)					
Baseline	1.63 ± 0.45	1.49 ± 0.33	1.69 ± 0.41	1.66 ± 0.32	1.79 ± 0.52	0.583
After 4 weeks	1.59 ± 0.40	1.36 ± 0.27 ^a^	1.65 ± 0.41	1.59 ± 0.24 ^a^	1.82 ± 0.52	0.229
After 8 weeks	1.54 ± 0.45	1.33 ± 0.27 ^a^	1.57 ± 0.39	1.53 ± 0.26 ^a^	1.74 ± 0.39	0.745
**Glucose (mmol/L)**						
Reference range	3.3–6.1 mmol/L					
Baseline	5.65 ± 0.49	5.48 ± 0.49	5.76 ± 1.22	5.28 ± 0.55	5.54 ± 0.46	0.499
After 4 weeks	5.35 ± 0.47	5.26 ± 0.47	5.50 ± 1.00	5.22 ± 0.39	5.46 ± 0.63	0.821
After 8 weeks	5.05 ± 0.38 ^a^	4.99 ± 0.58 ^a^	5.34 ± 1.02 ^a^	5.01 ± 0.38	5.06 ± 0.49 ^a^	0.637

ANOVA was performed to compare means at baseline or change at week 4 or week 8 from baseline between groups. *p* ^a^-value denotes significant (*p* < 0.05) difference from initial value within group determined by Student’s paired-samples *t*-test or Wilcoxon signed-rank test.

**Table 5:** Effects of dietary fibre supplementation during energy-restricted diet on inflammatory parameters.

	IP1 Mean ± SD	IP2 Mean ± SD	IP3 Mean ± SD	IP4 Mean ± SD	PP Mean ± SD	*p*
N (F/M)	17 (14/3)	15 (14/1)	16 (15/1)	17 (15/2)	15 (14/1)	
**INFLAMMATORY** **MARKERS**						
**CRP (mg/L)**						
Baseline	1.82 ± 1.31	2.25 ± 1.58	1.02 ± 0.38	1.54 ± 1.60	1.45 ± 0.98	0.345
After 4 weeks	1.71 ± 1.70	2.39 ± 1.97	1.16 ± 1.27	1.20 ± 1.03	1.40 ± 0.96	0.671
After 8 weeks	1.64 ± 1.35 ^a^	1.63 ± 1.50 ^a^	1.09 ± 0.69	1.22 ± 1.24 ^a^	1.68 ± 1.36	0.041 ^b^
**IL-6 (pg/mL)**						
Baseline	2.18 ± 1.07	4.51 ± 2.88	1.59 ± 0.26	1.61 ± 0.75	2.13 ± 1.45	0.293
After 4 weeks	2.96 ± 2.41	2.24 ± 1.16	1.49 ± 0.75	1.34 ± 0.66	1.98 ± 1.15	0.082
After 8 weeks	2.88 ± 1.52	2.97 ± 1.49	1.88 ± 0.59	2.49 ± 1.77	3.36 ± 3.77	0.395
**TNF-α (pg/mL)**						
Baseline	0.21 ± 0.06	0.23 ± 0.07	0.19 ± 0.05	0.21 ± 0.04	0.23 ± 0.07	0.861
After 4 weeks	0.17 ± 0.02	0.21 ± 0.03	0.17 ± 0.04	0.18 ± 0.04	0.22 ± 0.05	0.965
After 8 weeks	0.23 ± 0.07	0.19 ± 0.06	0.19 ± 0.04	0.20 ± 0.07	0.26 ± 0.09	0.689
**GUT PERMEABILITY**						
**LBP (µg/mL)**						
Baseline	10.6 ± 1.6	11.2 ± 4.1	10.2 ± 5.8	10.8 ± 1.3	11.4 ± 4.2	0.990
After 4 weeks	12.2 ± 2.0 ^a^	9.5 ± 4.9 ^a^	6.5 ± 6.0	7.5 ± 2.2 ^a^	9.7 ± 5.1	0.419
After 8 weeks	10.1 ± 2.0	12.9 ± 3.9	7.9 ± 4.1	11.9 ± 5.6	10.3 ± 5.0	0.501

ANOVA was performed to compare means at baseline or change at week 4 or 8 weeks from baseline between groups. *p* ^a^-value denotes significant (*p* < 0.05) difference from the initial value within group determined by Student’s paired-samples *t*-test or Wilcoxon signed-rank test; *p* ^b^-value denotes differences in change at week 4 or 8 weeks from baseline between groups determined by one-way ANOVA.

**Table 6:** Product evaluation.

	IP1	IP2	IP3	IP4	PP
Overall likeability	7.1	4.8	6.8	7.3	6.3
Taste acceptability	3.9	2.9	3.7	3.9	4.0
Texture acceptability	3.9	1.8	3.1	4.3	3.8

**Table 7:** Frequency of reported side effects and changes in appetite for each condition after 4 weeks of dietary fibre supplementation.

SIDE EFFECTS	IP1	IP2	IP3	IP4	PP
**Gastrointestinal discomfort**	15%	55%	20%	27%	-
Flatulence	15%	55%	20%	-	-
Stool change	-	15%	-	-	-
**Abdominal discomfort**	15%	13%	17%	7%	-
Bloating	-	-	-	-	-
Cramping	-	-	-	-	-
Feeling too full	15%	13%	17%	7%	-
Meal replacement	-	-	-	-	-
**Changed bowel habits**	-	13%	20%	27%	-
Urgency of bowel movements	-	-	-	-	-
Problems with defecation	-	-	20%	-	-
Dehydration	-	-	-	-	-
Nausea	-	-	-	-	-
Diarrhoea	-	6%	-	-	-
Constipation	-	13%	-	27%	-
**Easier bowel movement**	-	-	-	-	-
**DECREASED APPETITE**	15%	13%	17%	7%	-

**Table 8:** Dietary intake at baseline and after 8 weeks.

	IP1 Mean ± SD	IP2 Mean ± SD	IP3 Mean ± SD	IP4 Mean ± SD	PP Mean ± SD	*p*
**Energy intake (kJ/day)**						
Baseline ^a^	7734 ± 1651	8353 ± 1201	7938 ± 1785	7707 ± 1683	7504 ± 1715	0.991
After 8 weeks ^b^	6155 ± 728	6911 ± 2008	6148 ± 1176	6227 ± 1009	6485 ± 1079	0.998
**CHO intake (% E)**						
Baseline ^a^	43 ± 4	42 ± 13	43 ± 8	44 ± 13	44 ± 5	0.892
After 8 weeks ^b^	47 ± 4	50 ± 10	47 ± 6	45 ± 8	48 ± 6 ^c^	0.229
**Fat intake (% E)**						
Baseline ^a^	39 ± 3	37 ± 17	35 ± 14	36 ± 6	36 ± 8	0.945
After 8 weeks ^b^	30 ± 7 ^c^	32 ± 5	30 ± 6	30 ± 7	27 ± 7 ^c^	0.680
**Protein intake (% E)**						
Baseline ^a^	18 ± 5	19 ± 6	18 ± 5	19 ± 8	17 ± 5	0.975
After 8 weeks ^b^	22 ± 4 ^c^	21 ± 4	23 ± 5 ^c^	24 ± 6	24 ± 6	0.688
**Fibre intake (% E)**						
Baseline ^a^	21 ± 4	25 ± 5	23 ± 6	19 ± 5	19 ± 7	0.705
After 8 weeks ^b^	25 ± 6	28 ± 7	22 ± 6	20 ± 6	25 ± 5	0.922

Mean ± SD for dietary components at baseline and after 8 weeks with dietary fibre supplements or placebo product. The fibre content of the treatment products is not included. ANOVA was performed to compare the means between the groups at baseline ^a^ and the changes at week 8 ^b^. *p* ^c^-value denotes significant (*p* < 0.05) difference from the initial value within group determined by Student’s paired-samples *t*-test or Wilcoxon signed-rank test.

## Data Availability

Data will be made available upon reasonable request to the corresponding authors.
